# Structural insights into phenylethanolamines high-affinity binding site in NR2B from binding and molecular modeling studies

**DOI:** 10.1186/1756-6606-1-16

**Published:** 2008-11-18

**Authors:** Fui-Mee Ng, Matthew T Geballe, James P Snyder, Stephen F Traynelis, Chian-Ming Low

**Affiliations:** 1 Department of Pharmacology, Yong Loo Lin School of Medicine, National University of Singapore, Singapore; 2 Department of Chemistry, Emory University, Atlanta GA, USA; 3 Department of Pharmacology, Emory University School of Medicine, Atlanta GA, USA; 4 Neurobiology and Ageing Programme, Life Science Institute, National University of Singapore, Singapore

## Abstract

**Background:**

Phenylethanolamines selectively bind to NR2B subunit-containing *N*-methyl-*D*-aspartate-subtype of ionotropic glutamate receptors and negatively modulate receptor activity. To investigate the structural and functional properties of the ifenprodil binding domain on the NR2B protein, we have purified a soluble recombinant rat NR2B protein fragment comprising the first ~400 amino acid amino-terminal domain (ATD2B) expressed in *E. coli*. Spectral measurements on refolded ATD2B protein demonstrated specific binding to ifenprodil. We have used site-directed mutagenesis, circular dichroism spectroscopy and molecular modeling to obtain structural information on the interactions between critical amino acid residues and ifenprodil of our soluble refolded ATD2B proteins. Ligand-induced changes in protein structure were inferred from changes in the circular dichroism spectrum, and the concentration dependence of these changes was used to determine binding constants for ifenprodil and its analogues.

**Results:**

Ligand binding of ifenprodil, RO25,6981 and haloperidol on soluble recombinant ATD2B determined from circular dichroism spectroscopy yielded low-to-high micromolar equilibrium constants which concurred with functional IC_50 _measurement determined in heterologously expressed NR1/NR2B receptors in *Xenopus *oocytes. Amino acid residue substitutions of Asp101, Ile150 and Phe176 with alanine residue within the ATD2B protein altered the recombinant protein dissociation constants for ifenprodil, mirroring the pattern of their functional phenotypes. Molecular modeling of ATD2B as a clam-shell-like structure places these critical residues near a putative ligand binding site.

**Conclusion:**

We report for the first time biochemical measurements show that the functional measurements actually reflect binding to the ATD of NR2B subunit. Insights gained from this study help advance the theory that ifenprodil is a ligand for the ATD of NR2B subunit.

## Background

*N*-methyl-*D*-aspartate (NMDA) receptors are postsynaptic receptors for L-glutamate, the major excitatory neurotransmitter in the central nervous system. They are ligand-gated ion channels that are permeable to K^+^, Na^+ ^and Ca^2+^. They play a pivotal role in neuronal development, synaptic plasticity, and learning and memory [1-5]. However, the overactivation of these receptors has been implicated in excitotoxic neuronal cell death observed in neurological disorders such as stroke, epilepsy [6,7] and head trauma [8].

**Figure 1:**
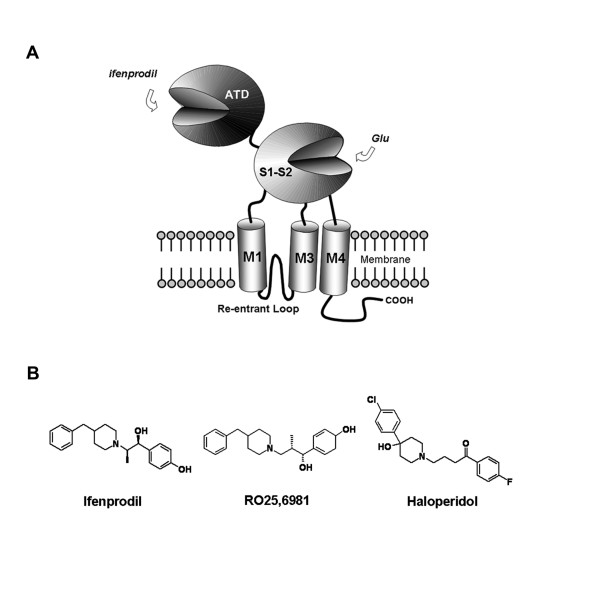
**Modular organization of NR2B subunit of NMDA receptor**. *A*. Diagram shows the amino-terminal domain (ATD), the two lobes (S1–S2) comprising the glutamate binding domain, the three transmembrane domains (M1, M3 and M4), the re-entrant loop and the intracellular cytoplasmic terminus (-COOH). ATD has been proposed to harbor the binding site for phenylethanolamines such as ifenprodil. *B*. Chemical structures of ifenprodil, RO25,6981 and haloperidol – ligands that bind to and negatively modulate the current flux through NR2B subunit-containing NMDA receptors.

NMDA receptors are heteromeric structures composed of an obligatory NR1 subunit and one or more of the four NR2 subunits (NR2A-D) and/or NR3 subunits (NR3A-B) [9-12]. Each NMDA receptor subunit shares a characteristic modular architecture with three transmembrane segments (M1, M3 and M4), a re-entrant loop (initially known as M2) which forms the pore-lining region, an intracellular C-terminal domain, and large extracellular domains [5,10,12]. The large extracellular domains of the mature NR2B subunit include amino acids 27–557 (Accession number U11419) situated pre-M1 and amino acids 649–817 between M3 and M4. The glutamate binding pocket is made up of two discontinuous stretches of amino acids encoded by the S1 and S2 regions of cDNA. Each segment constitutes ~150 amino acids preceding M1 and following M3. The remaining non-agonist binding segment preceding M1 is also known as the amino-terminal domain (ATD) (Fig. 1A).

Expression of isolated domains has been applied to ligand-gated glutamate receptors, voltage-gated potassium ion channels and many other plasma membrane bound ion channels and receptors with reasonable success. For NMDA receptors, the crystal structures of the ligand binding core (S1S2) for glycine (NR1) and glutamate (NR2A) have been successfully solved recently [13,14]. These atomic structures provide, for the first time, a bridge between data obtained from biophysical, molecular and biochemical studies of NMDA receptors. There are now over 50 crystal structures of various glutamate receptor binding cores bound to different ligands [15-19].

In contrast, there is no crystal structure for the putative allosteric modulators' binding sites located on the amino terminal domain proximal to the S1 region of ionotropic glutamate receptors, including NMDA receptors. The apparent high affinity Zn^2+ ^binding site is situated in ATD of NR2A [20-22]; the putative phenylethanolamines binding site(s) in ATD of NR2B [23-25]; the polyamine-sensitive region in ATD of NR1 [26,27]; and possibly a Zn^2+ ^binding site in NR2B [28]. Indeed, Rosahl and colleagues [25] provided *ex vivo *support for the modulatory role of ATD of NR2B by ifenprodil and ifenprodil-like NMDA receptor antagonists recently. By replacing the mouse NR2B gene function by "knocking-in" a chimeric human NR2A/B cDNA containing ATD of NR2A fused to the endogenous NR2B locus, the high affinity component from the inhibition of NR2B containing receptors was absent in culture hippocampal neurons.

Like the ATD of NR2A subunit of NMDA receptor which binds the negative allosteric modulator Zn^2+ ^[20-22,29], NR2B ATD binds negative modulator phenylethanolamines and analogues (ifenprodil, RO25,6981, haloperidol) (Fig. 1B) [23,24,30-34]. The NMDA receptor subunit ATD is a large domain made up of the first ~400 amino acids (e.g. residues 27–403) of the mature polypeptide. The ATD has weak homology and shows similarities in predicted secondary structure with bacterial periplasmic leucine/isoleucine/valine-binding protein (LIVBP) [27,35]. NMDA receptor ATD also shares homology and predicted secondary structure with the G-protein-coupled metabotropic glutamate receptor 1 (mGluR1), for which the crystal structure of the glutamate binding domain is known [36]. There is no crystallographic structural information on the ATD of NMDA receptor or any other ionotropic glutamate receptors at atomic resolution. This has slowed advances in our understanding about how the negative modulators inhibit the opening of the NMDA receptor channel, which is triggered by glutamate binding to the agonist binding core [37]. However, several low level electron microscopy studies suggest an orientation of ATD with respect to the glutamate binding core in AMPA receptors [38-40].

To begin to address this gap in our understanding, we have constructed and expressed the modular amino-terminal domain (proximal 367 amino acids) of NR2B subunit (ATD2B) as a 6xHis-fusion protein (6 × His-ATD2B) in *Escherichia coli *expression system [41]. In this study, we demonstrate, for the first time, that soluble 6 × His-ATD2B recombinant proteins can bind various modulators with dissociation constants (K_D_s) close to and correlated tightly with the functional IC_50 _measurements. Mutations at the critical amino acids (Asp101, Ile150, Phe176) within ATD of NR2B subunit significantly decrease the binding affinity of ifenprodil to 6 × His-ATD2B. Three-dimensional homology model of the ATD of NR2B revealed that these critical amino acids are strategically positioned around the putative ligand binding cleft. Taken together, our multi-disciplinary approach supports the concept of a modular organization of ATD within the NR2B subunit as well as its inherent ability to bind negative allosteric modulating ligands.

## Results

### Buffers -9, -12 and -13 refolded ATD of NR2B

Using a fractional factorial folding screen [41,42], we had successfully refolded 6 × His-ATD2B recombinant protein in three buffers differing in pH, refolding temperature, divalent ions, polar and nonpolar additives, chaotrope, GSH:GSSH ratio, detergent, PEG, and presence/absence of a ligand. These buffers were described in our previous work [see Additional file 1], and are referred to here by the same nomenclature as that study (Buffers -9, -12 and -13 [41]). We had shown previously Buffer-13 refolded 6 × His-ATD2B existed in a soluble and monodispersed species which bound ifenprodil in a concentration-dependent manner (K_D _= 128 nM) [41].

In this study we examine the structural and functional properties of refolded recombinant 6 × His-ATD2B proteins. Light scattering detection analysis allows biomolecular sizing and characterization of proteins to be measured directly. Dynamic light scattering analyses showed Buffers -9 and -12 refolded 6 × His-ATD2B proteins existed in monodispersed species (data not shown). Their relative percentages of α-helix content were estimated to be 26.7 ± 0.4% (n = 5) and 27.7 ± 0.7% (n = 6), respectively. These values were congruent with the 26.8% estimated for Buffer-13 refolded protein reported previously [41].

Circular dichroism (CD) spectroscopy is a useful technique for studying protein-protein interactions in solution [43]. Circular dichroism spectroscopy measures differences in the absorption of left-handed polarized light versus right-handed polarized light. Units given are typically ellipticity (theta, θ), which is related to the difference in absorbance of left and right circularly polarized light. This difference arises from the interaction between the light and the chiral nature of the protein structure. The chromophore for wavelengths in the "far-UV" spectral region (190–250 nm) is the peptide bond, and the signal arises when it is located in a regular, folded environment. Secondary structural elements such as alpha-helix, beta-sheet, and random coil structures each give rise to a characteristic shape and magnitude of CD spectrum. The CD spectrum of a protein in the "near-UV" spectral region (250–350 nm) can be sensitive to the overall tertiary structure of the protein, including signals for phenylalanine (250–270 nm), tyrosine (270–290 nm), and tryptophan (280–300 nm), as well as weak signals from disulfide bonds throughout the near-UV spectrum. Thus, evaluation of changes in the CD spectrum can be used to monitor changes in protein conformations following ligand binding, and the concentration dependence of ligand-induced changes in the CD spectrum can also be used to estimate binding affinities [44-46].

**Figure 2:**
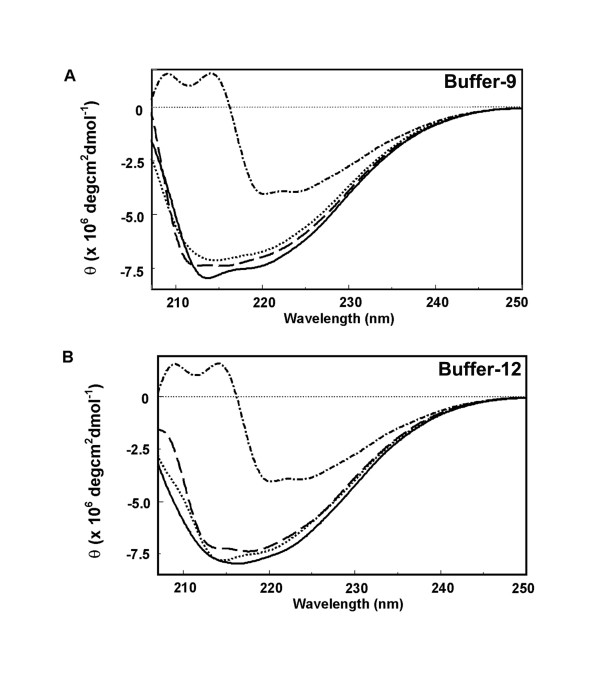
**Representative CD spectra of soluble 6 × His-ATD2B proteins in the presence and absence of ligands at 25°C**. *A*. Buffer -9 refolded protein; *B*. Buffer -12 refolded protein; Solid line, protein in absence of ligand; Dotted line, in presence of 5 μM ifenprodil; Dashed line, in presence of 0.5 μM RO25,6981; Dashed-dotted line, 4 M Gdn.HCl-denatured protein. Y-axis represents the molecular ellipticity. X-axis represents the range of wavelengths analyzed.

**Figure 3:**
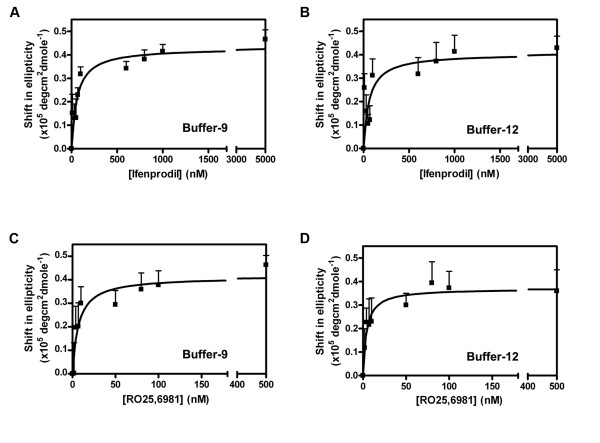
**Ifenprodil and RO25,6981 titration curves of soluble 6 × His-ATD2B proteins**. *A*. Buffer -9 refolded protein in the presence of ifenprodil; *B*. Buffer -12 refolded protein in the presence of ifenprodil; *C*. Buffer -9 refolded protein in the presence of RO25,6981; *D *Buffer -12 refolded protein in the presence of RO25,6981. The results are the average of five experiments using proteins obtained from at least two independent batches of bacteria induction. Error bars represent S.E.

**Figure 4:**
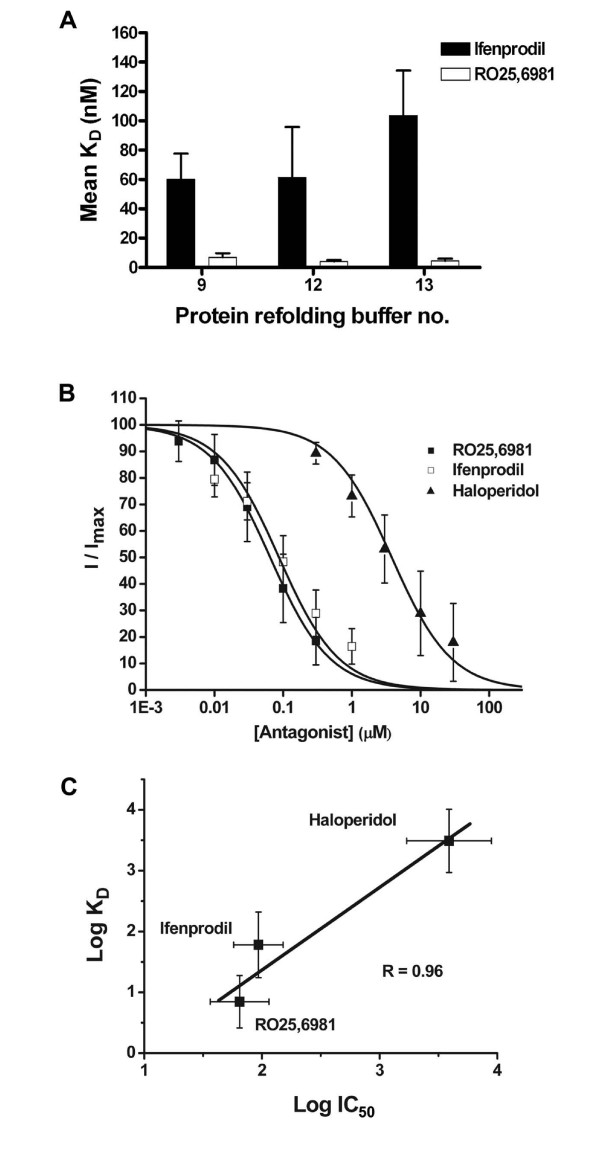
**Bar chart showing the relative affinities of ifenprodil and RO25,6981 towards the three buffers' refolded 6 × His-ATD2B proteins**. *A*. Results are the average of five experiments using proteins obtained from at least two independent batches of bacteria induction. Error bars represent S.E. Ratios of K_D_(ifenprodil) to K_D_(RO25,6981) are 9 (Buffer-9), 15 (Buffer-12) and 24 (Buffer-13), respectively. *B*. Composite antagonists' inhibition curves are shown for NR1 coexpressed with wild-type NR2B in *Xenopus *oocytes. Error bars represent ± SEM. *C*. The Log IC_50 _for RO25,6981, ifenprodil and haloperidol plotted against their respective Log K_D _values obtained from dose-titration using CD. A linear regression fit revealed good correlation (R = 0.96) between the mean Log IC_50 _and Log K_D _values for each ligand. The mean of the sum of individual log value obtained from each fitted dose-inhibition plot for each ligand and SEM are plotted. Mean K_D _and IC_50 _values are given in Table 1.

CD spectra of the Buffers -9 (Fig. 2A) and -12 refolded (Fig. 2B) 6 × His-ATD2B proteins were obtained in the presence of 5 μM ifenprodil and 0.5 μM RO25,6981. Ifenprodil and RO25,6981 in the concentration ranges 0.01–5 μM and 0.002–0.5 μM, respectively, diminished the θ over the wavelengths in the region 210–250 nm. We interpret the concentration-dependent shifts of magnitude at wavelength 220.0 nm ellipticity to suggest that ifenprodil binds to refolded soluble 6 × His-ATD2B proteins with K_D _values of 60 ± 18 nM for Buffer-9 (n = 5, Fig. 3A) and 61 ± 34 nM for Buffer-12 (n = 5, Fig. 3B). The concentration-dependent changes in the CD spectrum induced by RO25,6981 (218.9 nm) upon interacting with the 6 × His-ATD2B proteins suggest that the ligand binds with K_D _values of 7 ± 3 nM (Buffer-9), 4 ± 1 nM (Buffer-12) (n = 5, Figs. 3C, D) and 4 ± 2 nM (Buffer-13). Figure 4A summarizes the binding properties of 6 × His-ATD2B to ifenprodil and RO25,6981 between three different protein refolding buffers. In addition, in the presence of saturating concentration of 5 μM ifenprodil, RO25,6891 could displace ifenprodil and bind to 6 × His-ATD2B with comparable affinities in the absence of ifenprodil: Buffer -9 (4 ± 1 nM), Buffer -12 (2 ± 1 nM) and Buffer -13 (3 ± 1 nM).

### Ligand Inhibition of Heterologously Expressed NR1/NR2B Receptors in *Xenopus *Oocytes

Ifenprodil and RO25,6981 are NR2B-selective antagonists. Although both molecules share similar chemical structures that are likely to act on the same putative binding site residing in the ATD of NR2B, RO25,6981 is more potent than ifenprodil in inhibiting heterologously expressed and native NR2B-containing NMDA receptors (see [31] for review). We heterologously expressed NR1 with NR2B in *Xenopus *oocytes to determine the NMDA receptor sensitivity to both ifenprodil and RO25,6981 so as to establish the ratio of IC_50_(ifenprodil) to IC_50_(RO25,6981). This ratio will determine which buffer-refolded 6 × His-ATD2B (ie. Buffer-9 vs Buffer-12 vs Buffer-13) will be used for detail binding analyses. The negative allosteric antagonists inhibited NR1/NR2B receptor current responses with IC_50 _values 105 nM and 59 nM at pH7.3, respectively (Fig. 4B; Table 1). The ratio of IC_50_(ifenprodil) to IC_50_(RO25,6981) is almost 2.

**Table 1:** Binding properties of ligands to soluble 6 × His-ATD2B protein and their inhibition on heterologously expressed NR1-1a/NR2B receptors in *Xenopus *oocytes.

***Ligand***	***K*_*D*_*(nM)***		***IC*_50_*(nM)***	
RO25,6981	7 ± 3	(n = 5)	59 ± 1	(n = 22)
Ifenprodil	60 ± 18	(n = 5)	105 ± 9	(n = 20)
Haloperidol	3094 ± 940	(n = 5)	2762 ± 363	(n = 18)

Dose-titration binding constants of RO25,6981, ifenprodil and haloperidol performed on Buffer -9 refolded 6 × His-ATD2B protein determined from CD. Values are mean ± S.E. of the K_D _values from five experiments using proteins obtained from at least two independent batches of bacteria induction. Rat NR1 and NR2B subunits were coexpressed in *Xenopus *oocytes. NR2B-selective antagonists' inhibition of current responses induced by glutamate and glycine at pH7.3 were analyzed to obtain the mean-fitted IC_50 _values with n representing number of oocytes recorded.

### 6 × His-ATD2B Binds Antipsychotic Drug Haloperidol

6 × His-ATD2B refolded in Buffer-9 was able to bind a number of ligands with a wide range of affinities. Haloperidol, a dopaminergic antagonist with antipsychotic properties, is also known to block NR2B-containing NMDA receptors at micromolar concentrations [47-49]. Analysis of changes in CD spectra suggested that haloperidol bound to Buffer-9 refolded 6 × His-ATD2B in a dose-dependent manner. The maximum magnitude shift in ellipticity over 0.1–30 μM of haloperidol at 219.6 nm yielded a K_D _of 3094 nM (Table 1).

If the K_D _values estimated for the various ligands tested accurately represent dissociation equilibrium constants, then the biochemically determined K_D _values should correlate well with the IC_50 _values determined in functional assays. To address this, we performed linear regression analysis of three ligands with wide apart affinities (60–440 fold, Table 1; also see [48] and [34]) on our experimentally determined IC_50 _and K_D_. The ability of our Buffer-9 refolded 6 × His-ATD2B protein to bind three ligands of widely varying affinities (ifenprodil, RO25,6981 and haloperidol) yielded a tight correlation (R = 0.96; Fig. 4C). This strong linear correlation provided additional evidence that our soluble recombinant protein assumed a conformation that could bind its specific ligands.

Computing the ratios of K_D_(ifenprodil) to K_D_(RO25,6981) for each refolding buffers showed approximately 9-fold for Buffer-9, 15-fold for Buffer-12 and 24-fold for Buffer-13 refolded 6 × His-ATD2B proteins (Fig. 4A; Table 1). Buffer-9 refolded 6 × His-ATD2B yielded a ratio closest to the relative potency (IC_50 _ratio of 2) of ifenprodil to RO25,6981 (Fig. 4B and Table 1). Thus, subsequent structural and functional characterizations of the modular ATD of NR2B were performed on Buffer-9 refolded 6 × His-ATD2B protein.

### Impaired Binding Interactions between Recombinant ATD2B Mutants and Ifenprodil

**Figure 5:**
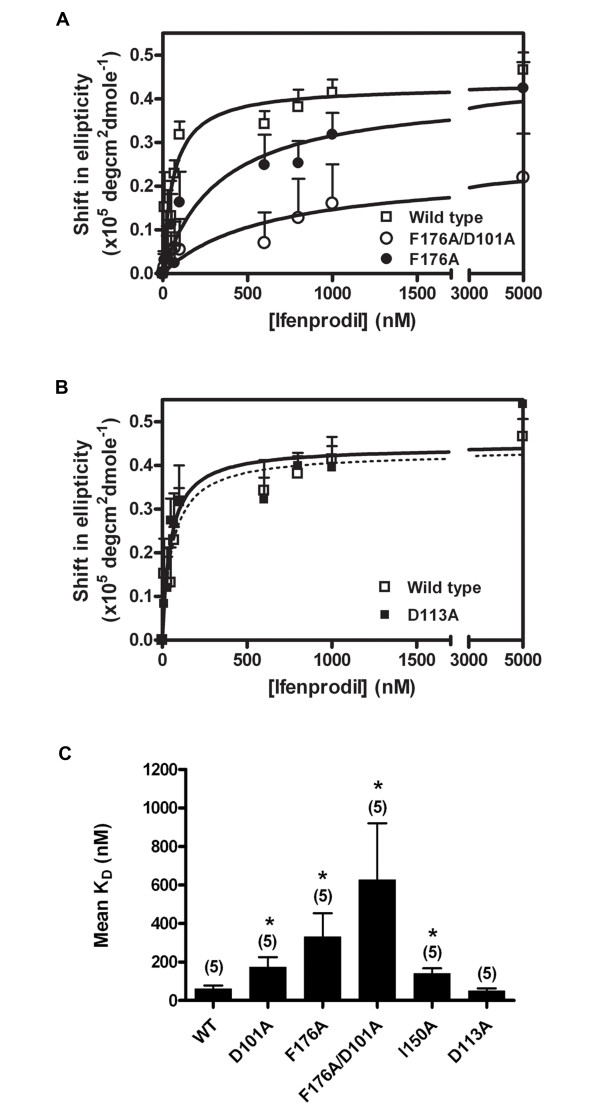
**Ifenprodil titration curve for Buffer -9 refolded wild type and mutant proteins**. *A*. Average dose-titration plots showing reduced affinity of ifenprodil to the F176A mutant protein (n = 5) as compared to the wild type protein (n = 5). The mutant protein harboring double mutations D101A/F176A (n = 5) (open circle) showed further reduction in affinity compared to the single mutant, F176A (filled circle). *B*. Mutation at residue Asp113, which was identified outside the putative ifenprodil binding pocket by our homology model, did not affect the binding affinity significantly (n = 5, P > 0.4). The K_D _values for different mutant proteins are listed in Table 2. Each data point is the average value obtained from five experiments using proteins obtained from at least two independent batches of bacteria induction. *C*. Mean-fitted K_D _values determined for WT protein and proteins containing the respective mutations. Paired Student t-Tests were performed to evaluate the difference between mean of K_D_(mutant) and K_D_(WT), * P < 0.05.

We next sought to more directly evaluate the ifenprodil binding pocket of Buffer-9 refolded 6 × His-ATD2B. A charged (Asp101), an aliphatic (Ile150) and an aromatic (Phe176) amino acid residues, previously reported to be critical molecular determinants for ifenprodil inhibition of NR2B-containing NMDA receptors (> 60-fold increase in IC_50 _values compared to wild-type NR1/NR2B receptors; [23]), were mutated to alanine (designated as ATD2B(D101A), ATD2B(I150A), ATD2B(F176A)) using site-directed mutagenesis (see Methods). We subjected these mutant 6 × His-ATD2B proteins to increasing concentrations of ifenprodil to evaluate the concentration dependence of binding. In the presence of increasing amounts of ifenprodil, each mutant protein D101A and I150A yielded higher K_D _values of 173 nM and 139 nM, respectively (Table 2). By substituting phenylalanine with alanine at amino acid 176, the concentration-effect curve yielded even higher K_D _value of 331 nM (Fig. 5A solid circle). When compared to the wild-type (WT) ATD2B protein, these mutations caused a 2.9- (D101A), 2.3- (I150A) and 5.5- (F176A) fold increase in K_D _values, which were statistically significant (P < 0.05) (Fig. 5C and Table 2).

**Table 2:** Comparison of K_D _values for the wild type and mutant 6 × His-ATD2B proteins.

**Protein**	**K_D _(nM)**	**Fold Shift**
WT	60 ± 18	1
F176A	331 ± 122	5.5
D101A	173 ± 52	2.9
F176A/D101A	627 ± 295	10.5
I150A	139 ± 28	2.3
D113A	48 ± 15	0.8

Values are mean ± S.E. of the K_D _values from five experiments using proteins obtained from at least two independent batches of bacteria induction. The fold shift in K_D _values for each mutant is the ratio of K_D_(mutant) to K_D_(WT).

We hypothesized that ifenprodil binding would destabilize further if more than one of the several key amino acid residues, whose side chain may make Van der Waals or hydrogen bonding contact with the ligand, were removed. We selected the charged Asp101 and hydrophobic Phe176 residues to disrupt jointly in our recombinant ATD2B protein. D101A was inserted into ATD2B(F176A) plasmid using overlap extension site-directed mutagenesis (see Methods). Indeed, this double mutant recombinant protein ATD2B(D101A/F176A) displayed a much weaker binding to ifenprodil (K_D _627 nM compared to 60 nM WT; P < 0.05; Fig. 5A, C; Table 2).

### Molecular Modeling of the Amino-Terminal Domain of the NR2B Subunit

**Figure 6:**
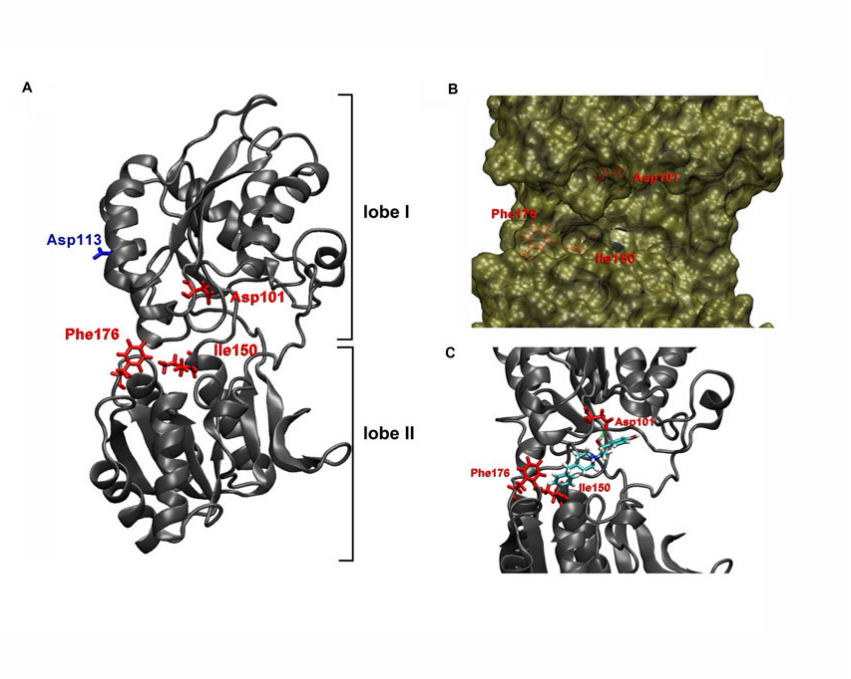
**Homology model of ATD of NR2B subunit**. *A*. Ribbon representation of the amino-terminal domain of NR2B was constructed as described under Materials and Methods using mGluR1 crystal structure (1 EWK) as template [36]. Model resembles the clam-shell configuration as seen in the bacterial periplasmic leucine-isoleucine-valine binding protein (LIVBP) (2 LIV). Critical residues (Asp101, Ile150, Phe176) that reduced ifenprodil sensitivity 60-fold and more, when mutated, as compared to wild-type NMDA receptors [23] are shown as red sticks using Rasmol. A mutation (D113A) outside the putative ifenprodil binding pocket is identified by blue sticks. *B*. Putative ifenprodil binding pocket highlighting the three critical amino acid residues that play critical role in interacting with ligand and the inhibition sensitivity of NR2B-containing NMDA receptors. *C*. Ifenprodil-docked into ATD2B putative binding pocket model. The relative orientations of side chains of Asp101 (top red residue in red sticks), Ile150 and Phe176 towards the putative centre cleft suggest plausible ligand-protein interactions with various functional groups on ifenprodil (see Discussion).

To better understand the structural determinants of ligand binding to ATD of NR2B, we used Prime Structure Prediction (Schrödinger) to build a homology model of ATD2B based on the crystal structure of mGluR1 agonist binding domain complexed with glutamate (1 EWK; [36]). The homology model of the ATD of NR2B in the apo (absence of ligand) configuration is depicted in Fig. 6A. It is built using the sequence alignment as shown in Additional file 2D. The α-helices and β-sheets were labeled according to the mGluR1 structure [36]. Three (Asp101, Ile150 and Phe176) out of four amino acids (Phe182) reported by Perin-Dureau and colleagues [23] that played a critical role in the inhibition of NR2B-containing NMDA receptors (IC_50_(mutant)/IC_50_(WT) > 60) are highlighted in Fig. 6A. Examinations of these three "critical" molecular determinants (D101A, I150A and F176A) reveal these residues to be spatially distributed near a central cleft despite their locations on different regions of the bi-lobed structure (Asp101 on lobe I while Phe176 on lobe II) as well as on the hinge region (Ile150) of the NR2B ATD (see also Fig. 8A in Perin-Dureau et al. [23]). Figures 6B and 6C show these three critical residues to be located within the putative central cleft and in contact with the top-scoring pose [50] of ifenprodil. In fact, ifenprodil is predicted to coordinate with Asp101 and Phe176 via hydrogen and hydrophobic interactions. In addition, the docked pose has significant Van der Waals contact with Val42, Thr103 and Lys234, residues that all had a moderate effect in previous studies [23] (data not shown).

Finally we searched for an amino acid residue located outside the cleft of the clam-shell conformation of NR2B ATD. A charged residue, Asp113 located on lobe I, was mutated and probed for its ligand binding phenotype (see Fig. 6A). The D113A mutation showed nearly superimposable dose-dependent ifenprodil titration curve with a K_D _similar to the wild-type soluble 6 × His-ATD2B (P > 0.4; Figs. 5B, C and Table 2).

## Discussion

In the present work, we have used an autonomously folded protein fragment of the amino terminal domain of NR2B subunit to elucidate several critical residues that influence the binding of putative modulators on the mammalian NR2B subunit-containing NMDA receptors. The two most important conclusions to emerge from this study are that (1) the isolated ATD2B protein is sufficient to interact with various modulators with affinities that correlate well with the physiological and biochemical studies, and (2) the critical molecular determinants for the binding of these modulators reside within the proximal ~400 amino acids preceding S1 domain of NR2B subunit. These data provide direct confirmation of ligand binding to ATD as well as suggest that mutations previously described influence the binding of ifenprodil, a NR2B-selective modulatory ligand.

### Comparison to previous studies

The K_D _values determined with CD spectroscopy of ligands for ATD2B correlated with functionally determined IC_50 _values (Fig. 4C), and were consistent with K_D _values previously reported for [^3^H]ifenprodil binding to rat brain cortex (K_D _= 36 nM) [51] and both NR1/NR2B transfected HEK293 and mouse L(tk-) cell line (K_D _= 34–79 nM) [52,53]. Furthermore, our ligand displacement experiment using RO25,6981 (~9-fold higher K_D _affinity determined in this study) over ifenprodil on ATD2B protein yielded K_i _values (2–4 nM for Buffers -9, -12 and -13, n = 5 per buffer; details not shown) comparable to K_D _values that were determined for RO25,6981 alone. Thus, our findings on refolded ATD2B agree well with previous determinations of ligand affinities determined in intact receptors.

Previous description of alanine mutagenesis scan in the NR2B ATD on key amino acid residues known to coordinate ifenprodil and RO25,6981 binding [23,24] corroborated our structural findings. Perin-Dureau and colleagues [23] showed that the substitutions of Asp101, Phe176 and Phe182 produced the largest effects on ifenprodil potency for inhibition of NR1/NR2B currents, and hypothesized that these residues were involved in ifenprodil binding. They further proposed that Ile150 participated in the clam-shell closure mechanism rather than direct contact with the ligand. D101A and F176A mutations inserted individually into the full length NR2B subunit of the heterologously expressed NR1/NR2B receptors yielded > 60-fold increase in IC_50 _values [23]. Our experimentally determined K_D _values for these two same mutations inserted into our ATD2B refolded protein were 2.3–5.5 fold higher than those determined from wild-type ATD2B. Our results are consistent with the ideas presented by Perin-Dureau et al. [23], and suggest that these residues can directly impact ifenprodil binding. However, the single residue mutant ATD2B proteins did not generate the same degree of shift in K_D _as observed for the IC_50 _value determined from two electrode voltage clamp current measurements. The IC_50 _values determined from the voltage clamp experiments describe the concentration-dependence of ifenprodil reduction in cation flux through the aqueous channel pore. This functional measure thus takes into account the effects of mutations on allosteric interactions between domains, long range intra-protein interactions, in addition to effects on ligand binding site. It seems likely that some of the differences in our K_D _and functional IC_50 _values are attributed to the study of ATD2B in isolation.

Our K_D _values, determined via dose-dependent ligand-binding assay using CD, are 'snap-shots' physical constants between the ligands and the isolated ATD protein. Interestingly, D101A and F176A mutations resulted in complete loss of [^3^H]RO25,6981 binding to membranes of HEK293 cells transiently co-transfected with NR1 and NR2B cDNAs [24]. Since both ifenprodil and RO25,6981 only differ chemically by a methylene between the piperidine ring and the phenyl ring (see Fig. 1B), one plausible scenario would be that other surrounding charged (eg. Asp104) and hydrophobic (eg. Phe182) residues could possibly compensate partially for the substituted Asp101 and Phe176, respectively [23]. Alternatively, both ligands may dock themselves in an opposite manner into the binding pocket within ATD2B. Notwithstanding these discrepancies, our experimental data on double-mutant ATD2B(D101A/F176A) recombinant protein, as predicted, yielded larger shift in its K_D_. The degree of this reduction in affinity for the double-mutant protein in association with ifenprodil is more than an additive effect when compared to single amino acid substitutions of D101A or F176A.

### Molecular features of ifenprodil binding and inhibition

Our homology model shows a clam-shell configuration of NR2B ATD with a central cleft. The carboxyl of Asp101 and the hydrophobic phenyl ring of Phe176, from lobe I and lobe II respectively, are located within the microenvironment of the cleft (Fig. 6B). The carboxyl group of Asp101 can potentially interact with the basic nitrogen on the piperidine ring, or either of the hydroxyl groups of ifenprodil via electrostatic interactions or hydrogen bonding. The phenyl rings of Phe176 and ifenprodil can establish hydrophobic interaction (Fig. 6C). The predicted distance between these two residues from our model is 11.2 Å (γ-carbon to γ-carbon). In the homology model, Ile150 is located at the end of one of three flexible loops that link Lobe I and Lobe II of the ATD. Together with other critical residues such as Phe176 and Phe182, its alkyl side chain helps form a hydrophobic backing to the pocket where ifenprodil and RO25,6981 are predicted to dock (Fig. 6C). As such, it is plausible to hypothesize that Ile150 and Phe182 potentially provide contact to ligands through nonspecific lipophilic interactions [23]. Ile150 and Phe182 may also provide a hydrophobic environment that helps organize other nearby residues such as Phe231 or Val258, or they may provide a limit on the length of the ligand (the phenol end) as McCauley and colleagues observed of large non-polar hydrophobic side chains larger than methyl at the phenol end of ifenprodil-like compounds, which have poor inhibitory properties on NR1/NR2B expressing cell lines [53]. In contrast to elongated binding poses with the ligand stretched across the cleft as seen in Marinelli et al. [54], the formation of a deep pocket allows the ligand to bury some of its hydrophobic regions as well as bringing it into proximity with other residues shown to be critical for binding.

## Conclusion

We have provided evidence for the binding of ifenprodil and its analogues directly to soluble recombinant ATD protein of NR2B subunit. We have also provided data supporting the direct involvement of intra-cleft residues predicted by a homology model of ATD2B in ifenprodil binding and developed a proposed binding mode that more clearly demonstrates the role of these residues. These show that the soluble folded ATD2B protein provides a new tool for structure-activity studies on existing and new subunit selective modulators that bind to glutamate receptor amino terminal domains [55].

## Methods

### Materials

Rat cDNAs for NR1-1a (hereafter NR1) and NR2 subunits (GenBank numbers NR1 U11418 and U08261; NR2B U11419) were provided by Dr. S. Heinemann (Salk Institute, La Jolla CA). The vector pET30b(+)EG was a gift from Dr E. Gouaux (Vollum Institute, Portland OR). Ifenprodil, RO25,6981 and haloperidol were obtained from Sigma. Oligonucleotides were purchased from Metabion (USA) and Proligo (Singapore).

### Site-directed mutagenesis

Residues that are targeted by mutagenesis in ATD sequence of NR2B subunit are shown in Additional file 2A. These amino acids were mutated in pET30b(+)EG-ATD2B plasmid [41] by overlap extension site-directed mutagenesis (D101A, I150A, F176A) [56,57] and QuikChange methods (D113A) [22]. The mutagenesis primers used are listed in Additional file 3. For overlap extension mutagenesis, two addition primers (2B-PRM5 and 2B-PRM6) [41] were coupled to the above mutant primers to first generate two amplicons with overlapping ends. The authenticity of the full-length cloned inserts in all recombinant constructs was confirmed with dideoxy DNA sequencing in both directions.

### Wild-type and Mutant 6 × His-ATD2B Protein Expression, Solubilization, Purification and Refolding

The wild-type and mutant 6 × His-ATD2B recombinant proteins were expressed, lyzed, solubilized, affinity-purified and refolded as described previously [41] [Additional file 2B, C]). All proteins were finally dialyzed against Buffer 2 [see Additional file 1]. The protein concentrations of purified 6 × His-ATD2B were determined by Bradford protein assay and absorbance at an extinction coefficient (ε_280 _= 60,430 M^-1^cm^-1^) as reported previously [41].

### Dynamic Light Scattering (DLS), Circular Dichroism (CD) Spectroscopy and Ligand-Binding Assay

DLS measurements were made with DynaPro (Protein Solutions) and CD spectra were measured at 25°C on a Jasco J-715 Spectropolarimeter equipped with a 0.1 cm path-length cuvette (Jasco Corporation, Tokyo, Japan) as described previously [41]. For the CD assays, the refolded protein was concentrated to 1 mg/ml using Vivaspin 2 ml concentrator (Vivascience). Ligand (haloperidol, ifenprodil or RO25,6981) was added in small aliquots from stock solution to 350 μl of the protein solution. The final change of assay volume was < 3% for all ligands investigated. The CD spectra of the wild-type and mutant 6 × His-ATD2B proteins in the absence and presence of a ligand were recorded from 190 to 250 nm. The CD spectrum of Buffer 2 was subtracted from all the CD spectra obtained. To ensure that the ligand molecules themselves did not cause changes in the molecular ellipticities, control experiments were performed before the binding assays whereby ligand was added into Buffer 2 and scanned from 190 to 250 nm. CD spectra of all ligands tested in this study did not give rise to noticeable absorbance (data not shown). All experiments were performed using at least two separate batches of induced and refolded 6 × His-ATD2B proteins (both wild-type and mutants), each batch in duplicates or triplicates. The dose-dependent binding curves of ligands to 6 × His-ATD2B were fitted by the following equation:

*Y *= *B*_*max *_[*ligand*]/(*K*_*D *_+ [*ligand*])

where *Y *is the shift in ellipticity, *[ligand] *is the concentration of ligand investigated and *K*_*D *_is the dissociation equilibrium constant.

### Electrophoresis and Mass Spectrometry

The wild-type and all mutant recombinant 6 × His-ATD2B proteins were separated, detected by specific antibodies recognizing hexahistidine tag (Clontech; 1:10,000) and N-terminal amino acids 27–76 of the NR2B subunit (Santa Cruz Biotechnology, Inc; 1:500). Detection of the primary antibody was performed with anti-rabbit IgG horseradish peroxidase (HRP) (1 mg/ml) followed by ECLPlus Western blotting detection reagents (Amersham Pharmacia, USA). Molecular weights of proteins were confirmed by MALDI-TOF MS analysis (Voyager DE-STR, Applied Biosystems). Trypsin-digested peptides of 6 × His-ATD2B were analyzed on 4700 Proteomic Analyzer (Applied Biosystems) and Data Explorer software [41].

### Voltage-Clamp Recordings from Xenopus Oocytes

cRNA for rat NR1-1a (hereafter NR1) and NR2B were synthesized *in vitro *and injected (5–10 ng) into stage V-VI *Xenopus laevis *oocytes, isolated as previously described [22,26,58]. Two-electrode voltage-clamp current recordings were made 2–7 days post injection. The recording solution contained (in mM) 90 NaCl, 3 KCl, 10 HEPES, 0.5 BaCl_2_, 0.01 EDTA, 0.05 glycine (23°C); pH was adjusted to 7.3 with NaOH. EDTA (10 μM) was added to chelate contaminant extracellular divalent ions, including trace amounts of Zn^2+^. Solution exchange was computer controlled through an 8-modular valve positioner (Digital MVP Valve, Hamilton Company, Reno, NV). Voltage and current electrodes were filled with 0.3 and 3.0 M KCl, respectively, and current responses recorded at a holding potential of -40 mV. Data acquisition and voltage control were accomplished with a two-electrode voltage-clamp amplifier (OC-725, Warner Instruments, Hamden, CT). Data were pooled among oocytes and mean composite concentration-effect curve was fitted by the following equation:

*Percent response *= 100 - *minimum*/(1 + ([*antagonist*]/*IC*_50_)^*n*^) + *minimum*

where *IC*_50 _is the concentration of antagonist that produces a 50% inhibition, *[antagonist] *is the concentration of antagonist investigated, *minimum *a residual current response fixed to 0 and *n *is the Hill slope.

### Homology Modeling and Docking

The protein sequence of the rat NR2B ATD (GenBank accession number U11419) was aligned against that of mGluR1 ATD (1 EWK; (36)) using a previously published alignment [24] [Additional file 2D]. Both the open and closed forms of mGluR1 were used as a structural basis for homology models, but due to recent work the closed model was used as the basis for docking [54]. The homology model was then built from this modified alignment using Prime (Schrödinger), and then subjected to multiple iterative rounds of energy minimization and side chain optimization to relieve any steric strain present in the model. Ifenprodil was constructed in Maestro (Schrödinger) and docked into the homology model using Glide (Schrödinger) and induced fit docking, with the box centered on residues Asp101, Asp104, Ile150, Phe176, Phe182, and Thr233. Docking was constrained by required contact with a previously identified critical residue (Asp101) [23]. These results were then rescored using the MM-GBSA protocol, which incorporates solvation and protein relaxation.

### Data Analysis

The shifts in ellipticities at 218.9 nm (RO25,6981), 219.6 nm (haloperidol) and 220 nm (ifenprodil), where the signal change on ligand binding was maximal, were calculated. The experiment data were analyzed by nonlinear curve fitting (ORIGIN7, USA). All pooled data were expressed as mean ± SEM. The numbers of CD assay for wild-type and mutants as well as the number of oocytes recorded on heterologously expressed NR1/NR2B receptors are shown in parentheses.

## Abbreviations

NMDA: N-methyl-D-aspartate; ATD: amino-terminal domain; LIVBP: leucine-isoleucine-valine-binding protein; DLS: dynamic light scattering; CD: circular dichroism; K_D_: dissociation constant.

## Competing interests

The authors declare that they have no competing interests.

## Authors' contributions

FMN carried out the recombinant protein biochemical studies. MTG carried out and JPS participated in the homology modeling and docking studies. SFT participated in the functional oocyte studies. CML conceived of the study, participated in its design and coordination, and drafted the manuscript. All authors read and approved the final manuscript.

## Supplementary Material

Additional file 1 **Refolding buffer compositions and dialysis temperatures of 6 × His-ATD2B recombinant protein.** Click here for file

Additional file 2 **Construction and expression of ATD of NR2B subunit**. A. Sschematic representation of the ATD2B construct (Arg27 to Arg393)  with 6xHis-tag and linker. Two stop codons are inserted (**). The four  mutated residues that were investigated in this study are labeled. B.  Coomassie blue-stained 11% SDS-PAGE gel showing insoluble protein  fraction induced with 0.5mM IPTG (lane 1), soluble protein fraction  (lane2) and purified protein (47kDa) after elution with 300mM imidazole  (lane 3). C. 6xHis-ATD2B probed with anti-6xHis (left) and anti-NR2B  (right) primary antibodies. D. Rat NR2B and mGluR1 ATD sequence  alignment (CLUSTAL W 1.83) and secondary structure assignment based on  mGluR1 [36]. Helices are shown as red cylinders, strands as blue arrows,  with the same labeling as in Fig. 2 of [36]. Intraprotomer disulphide  bridges identified in mGluR1 are indicated by thin solid black lines  connecting cysteines. Red amino acid residues indicate selected critical  molecular determinants to ifenprodil binding within ATD of NR2B which  are mutated in this study (also see [23] for more key residues); blue  amino acid residue indicates non-critical residue to ifenprodil binding.  Residues that are identical between mGluR1 and NR2B are shaded black  while conserved residues are shaded grey. Click here for file

Additional file 3 **Oligonucleotide primers used in mutagenesis experiments**. T_A _represents the annealing temperature. The nucleotides coding for the mutant amino acid are underlined. Click here for file
