# Supplementary figures and images for: Structural insights into phenylethanolamines high-affinity binding site in NR2B from binding and molecular modeling studies
Source: Mol Brain. 2008 Nov 18;1:16. doi: 10.1186/1756-6606-1-16 (PMC2603005; doi:10.1186/1756-6606-1-16)

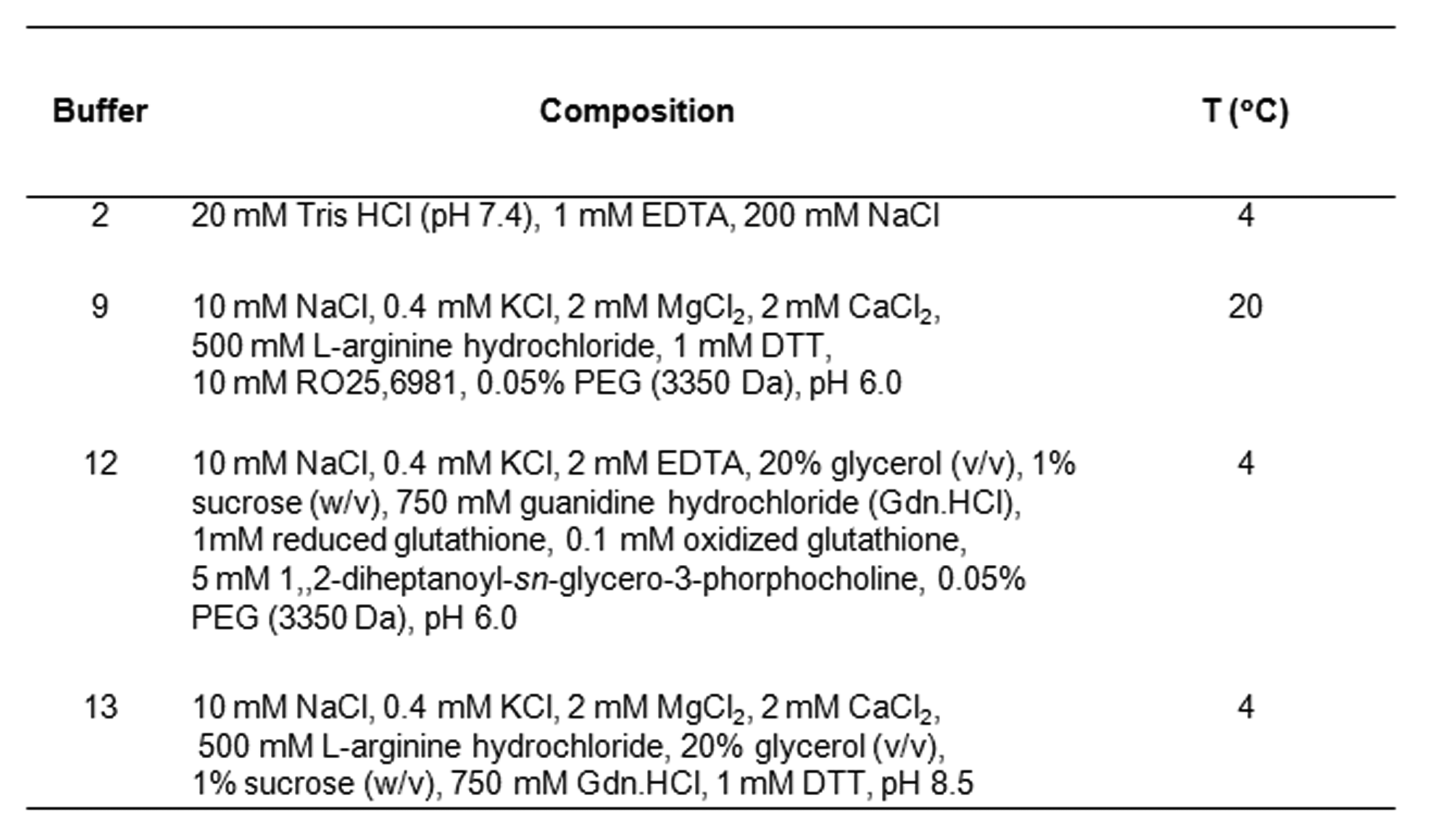

Supplement: Additional file 1 — Refolding buffer compositions and dialysis temperatures of 6 × His-ATD2B recombinant protein. [file 1756-6606-1-16-S1.tiff]

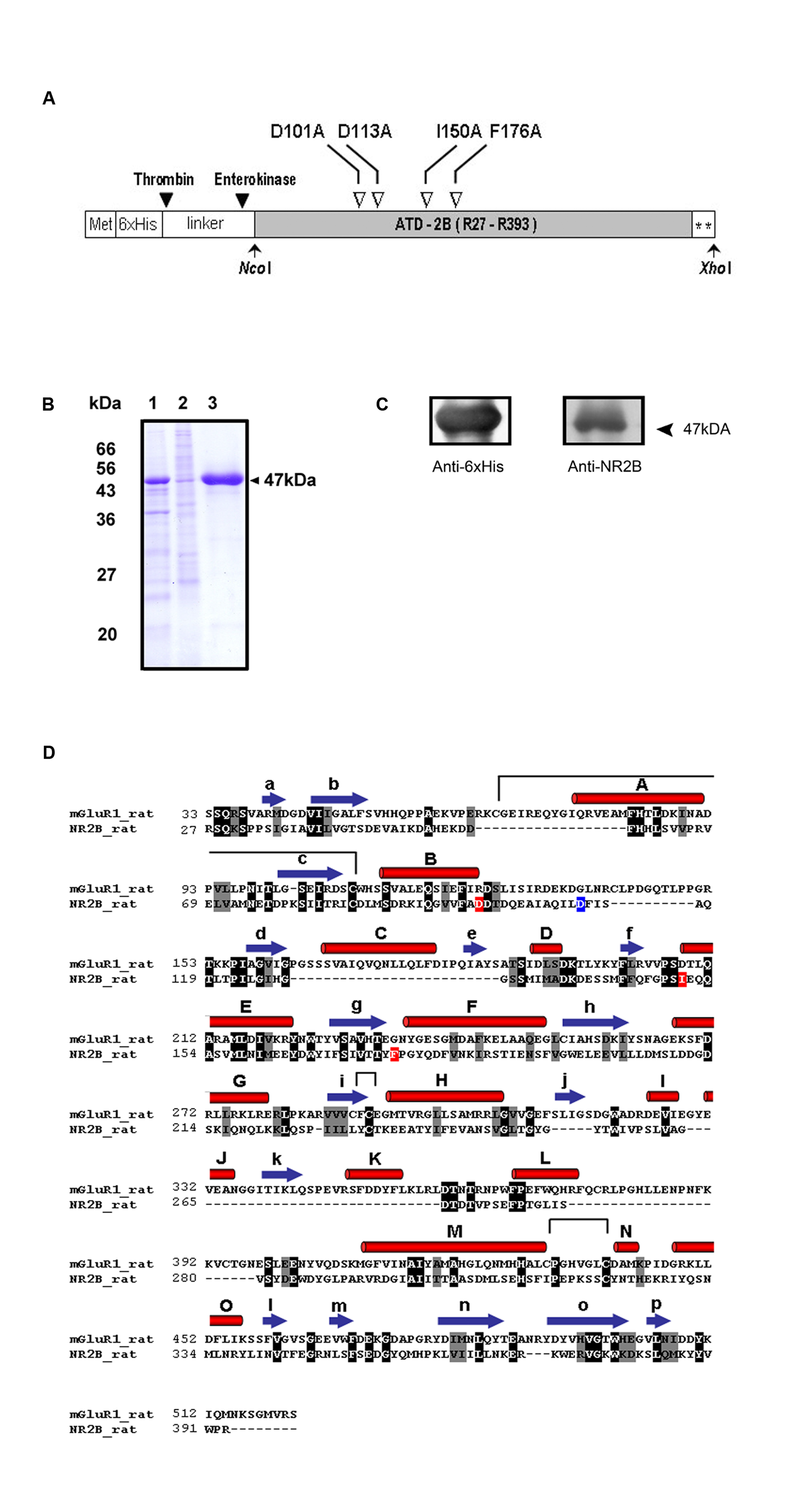

Supplement: Additional file 2 — Construction and expression of ATD of NR2B subunit. A. Sschematic representation of the ATD2B construct (Arg27 to Arg393) with 6xHis-tag and linker. Two stop codons are inserted (**). The four mutated residues that were investigated in this study are labeled. B. Coomassie blue-stained 11% SDS-PAGE gel showing insoluble protein fraction induced with 0.5mM IPTG (lane 1), soluble protein fraction (lane2) and purified protein (47kDa) after elution with 300mM imidazole (lane 3). C. 6xHis-ATD2B probed with anti-6xHis (left) and anti-NR2B (right) primary antibodies. D. Rat NR2B and mGluR1 ATD sequence alignment (CLUSTAL W 1.83) and secondary structure assignment based on mGluR1 [36]. Helices are shown as red cylinders, strands as blue arrows, with the same labeling as in Fig. 2 of [36]. Intraprotomer disulphide bridges identified in mGluR1 are indicated by thin solid black lines connecting cysteines. Red amino acid residues indicate selected critical molecular determinants to ifenprodil binding within ATD of NR2B which are mutated in this study (also see [23] for more key residues); blue amino acid residue indicates non-critical residue to ifenprodil binding. Residues that are identical between mGluR1 and NR2B are shaded black while conserved residues are shaded grey. [file 1756-6606-1-16-S2.tiff]

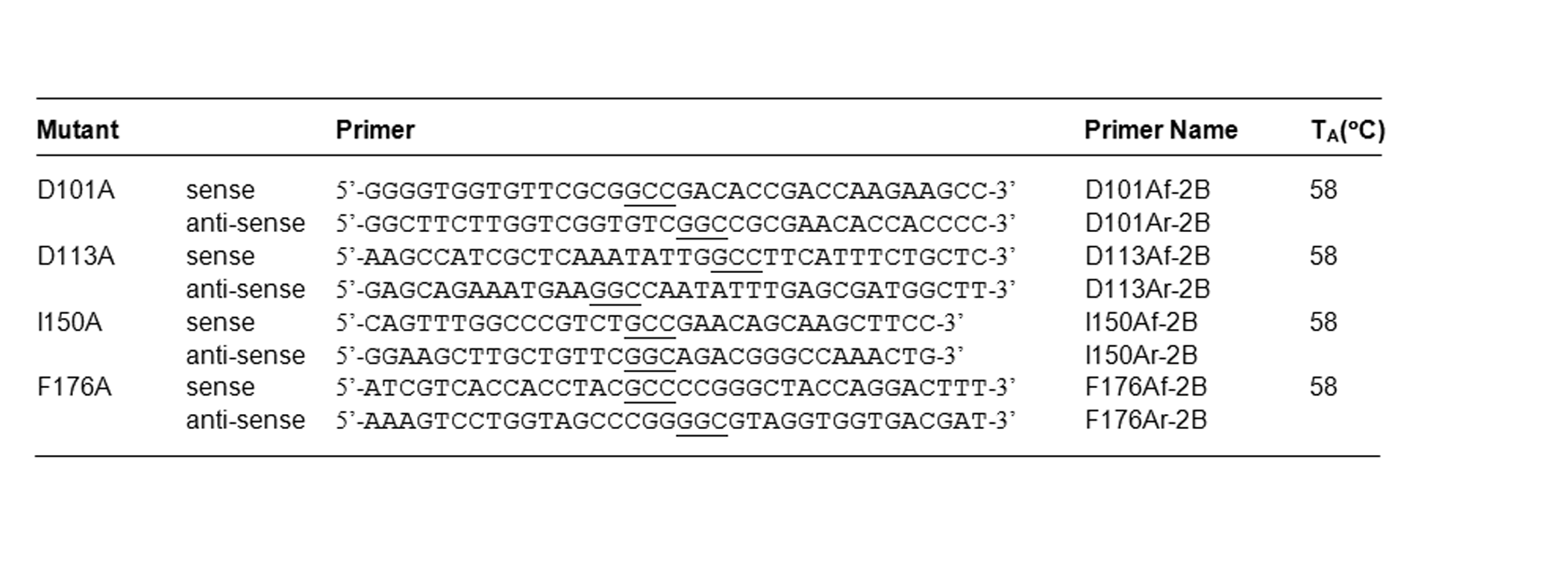

Supplement: Additional file 3 — Oligonucleotide primers used in mutagenesis experiments. TA represents the annealing temperature. The nucleotides coding for the mutant amino acid are underlined. [file 1756-6606-1-16-S3.tiff]
